# Dr. Samuel Russell Welles

**Published:** 1900-08

**Authors:** 


Dr. Samuel Russell Welles, U. of B. '48, of Waterloo, N. Y., died at his home in that village, July 15, 1900, aged 75 years. He was born at Waterloo, February 23, 1825, received the degree of A. B. at Geneva (now Hobart) College in 1845, and graduated in medicine at the University of Buffalo in 1848. He was appointed assistant surgeon in the 61st N.Y. Vols., March 8, 1862, and resigned his commission, July 25, 1862. This regiment was first commanded by Colonel (afterward Major General) Francis C. Barlow, and next by Colonel Nelson A. Miles, now Lieutenant-General and Commander-in-Chief of the Army of the United States. In 1875 Dr. Welles was elected a trustee of Hobart College and he has served two terms in the legislature, representing Seneca County.

